# It is Time to Realize the Promise of the Digital Mental Health Transformation: Application for Population Mental Health

**DOI:** 10.2196/63791

**Published:** 2025-06-19

**Authors:** Jonathan Adler, Deryk Van Brunt

**Affiliations:** 1 University of Massachusetts Chan Medical School, 55 N Lake Ave, Worcester, MA, 01655, United States, 1 (508) 856-8989; 2 School of Public Health, University of California, Berkeley, Berkeley, CA, United States

**Keywords:** mental health, mental illnesses, mental disorders, population mental health, population health, digital mental health, e-mental health, electronic mental health, digital, digital health, digital technology, digital interventions, digital health tools, mental health tools, mental health interventions

## Abstract

The past 25 years have seen the explosion of digital health care—from 1s and 0s initially serving most researchers for accomplishing their work, to the creation of smartphones, mHealth, and more recently artificial intelligence. The revolution for digital mental health is no longer in its infancy, as new tools are created to address mental health, sometimes even undergoing evaluation for adoption and efficacy. In fact, a recent study reporting on National Health Interview Survey data (annually conducted by the National Center for Health Statistics) indicated that, in 2024, 40% of adults reporting serious psychological distress used a digital health tool, which has increased from 21% in 2017 and 10% in 2013. Given the widespread access to digital tools and the potential of digital mental health, it is time for a new paradigm of care to address the mental health crisis in the United States. Reactive care, consisting largely of medication and counseling provided to those already experiencing severe or debilitating symptoms of mental anguish, is not adequate to address the needs of 22.8% of the US population (>55 million people) experiencing symptoms of a mental illness, and the larger number of people with preclinical mental health concerns. A population mental health approach is needed that includes early identification, intervention, and prevention, in addition to reactive care.

## Background

The US Substance Abuse and Mental Health Service Administration reports that the prevalence of mental illness has increased over the period from 2008 to 2020 [1]. Globally, suicide accounts for 726,000 deaths annually [2] and 48,183 deaths in the United States in 2021 [3]. The long-standing mental health crisis in the United States has only worsened in the past several years, with higher rates of anxiety, depression, and suicide in the decade preceding the SARS-CoV-2 pandemic [4-7], and with a further rise since [8]. The financial impact of mental and substance use disorders has increased along with this rise, with spending increasing from $171.7 billion in 2009 to $280.5 billion in 2020 [9]. Clinically relevant anxiety serves as an example of the challenges faced by both clinicians in making a diagnosis and by individuals in need of care. More than 40 million adults in the US experience a clinical anxiety disorder [10], and only 1 in 10 of these receive appropriate treatment [11]. Individuals experiencing anxiety and depression often suffer for years before getting treatment. Though nearly one-third of the people access care within the first year of their symptoms, the median times for access are 6‐8 years for mood disorders (such as depression) and 9‐23 years for anxiety disorders [12].

The stigma surrounding mental health remains a major barrier for people exploring their mental well-being and contributes to the delays in seeking treatment. Certain groups also face certification or employment repercussions associated with reporting mental health issues or seeking care. For example, a 2019 survey of 15,181 physicians found that about 25% (3795/15,181) had considered or attempted suicide; among these, 61% (9260/15,181) reported they have not and do not plan to seek help. The fear of disclosure is cited as the cause for not seeking care by 22% (3340/15,181) of responding physicians [13]. Just addressing stigma has been shown to be useful. A 2020 Deloitte study of workplace well-being found that an intervention that solely promoted mental health awareness had a return-on-investment of 7.5:1 [14].

Compounding these challenges is the significant shortfall of clinicians available to treat those with a mental health concern. The US Department of Health and Human Services estimates a shortfall of 126,830 direct mental health caregivers, and an additional shortage of 127,000 school counselors and mental health and substance abuse social workers, by 2025 [15]. The distribution of caregiver shortage is not equitable. Many rural areas have severe shortages. For example, overall, 51% (1603/3144) of counties in the US have no psychiatrist, and the shortage increases to 65% (2044/3144) in rural areas. In rural areas, 47% (1478/3144) of counties do not have even one psychologist [16].

The Health Resources and Services Administration reports that, in 2022, there are 53 counties with a total population of about 775,000 in North Dakota. A total of 54% (418,500/775,000) of North Dakotans have 0‐5 psychiatrists in their county, and 46 counties have none [17]. Over half of the US population, about 169 million people, live in a mental health care health professional shortage area [18].

Recognizing the critical need for the identification of those with mental health concerns, in 2023, the US Preventive Services Task Force, a major nongovernmental agency that often guides health care screening practices, recommended routine anxiety screening of all adults aged 19‐64 years [19]. Though this proposal represents progress, it falls short of what is needed in many respects. Most importantly, only patients scoring in the “severe” range of symptoms will be offered care. There is no path for intervention for the majority of patients who have mild to moderate, still symptomatic, anxiety. An equivalent in population medicine would be identifying patients with prediabetes, but offering no education or intervention until they require insulin injections. While this long-overdue triage for early detection and intervention is welcomed for anxiety, broader screening for other mental health concerns is needed. Furthermore, the current landscape of screening and reactive care falls woefully short of addressing high prevalence, subclinical mental health risk factors such as burnout, loneliness, languishing, or lack of purpose in life that are predictive of or correlated with stress, anxiety, and depression and are amenable to early intervention.

## Four Building Blocks of a Proactive Approach to Population Mental Health

### Background

The current “reactive care” model treats only those with an advanced stage of mental anguish and requires intensive interventions that are often insufficient and impractical due to caregiver shortages, especially in underserved areas. Intervention is needed upstream, before a person experiences severe negative symptoms consistent with a clinical diagnosis, has exhausted coping mechanisms, has lost productivity, or may be contemplating self-harm.

A population mental health approach integrating broad screening with evidence-based resources holds the potential to bolster mental health, enhance productivity, and curtail the burden and harms of reactive care. Furthermore, efforts to add early intervention and prevention fit well with the fact that about 75% of people with a mental health or substance use concern prefer to work on the issue on their own [20].

This represents an enormous opportunity to provide psychoeducation and evidence-based resources for self-management to this majority. A recent meta-analysis of 35 research papers studying the return-on-investment of early mental health intervention found cost savings, stating “In adults, strong evidence supported screening plus psychological interventions for mental disorder prevention, while workplace interventions targeting employees in general were cost-effective [21].”

A comprehensive public health framework, akin to successful approaches in addressing other health crises, can mitigate mental health challenges by reducing stigma, improving awareness, and providing accessible self-care in addition to reactive clinical support. This framework includes the following components: digital access, community screening, evidence-based self-care, and connection to screening-informed higher levels of care.

### Digital Access

Leveraging digital health technology facilitates large tracts of population accessing assessments, resources, and when needed, higher levels of care. The Pew Research Center reports that 93% of adults in the US use the internet [22]. Digital access has improved dramatically among groups with the highest rates of mental health concerns. Access among those earning less than US $30,000/year has climbed to 86%. Similarly, access in rural areas has risen to 90% [22]. Eighty-five percent of US adults own a smartphone [23]. Thus, web-based, smartphone-accessible platforms have the greatest potential to facilitate connecting large swaths of our population to screening and resources.

### Community Screening

Broad screening not only allows the early identification of those with symptomatic mental health concerns, it also intrinsically helps reduce stigma. Screening has been an effective tool in physical medicine not only for its direct benefit, but also for increasing awareness. Broad screening can be instituted as a component of proactive employer, community, caregiver, or member outreach programs.

### Evidence-Based Self-Care

Evidence-based self-care can be matched to personal behavior change and learning preferences. Early intervention and self-help can be effective approaches to treat or prevent a range of mental health concerns in many cases, with as good outcomes as effective as therapist-administered treatments [24-34].

Some examples include mindfulness-based stress reduction and early intervention.

Mindfulness-based stress reduction can be as effective as medication to treat anxiety. A randomized clinical trial published in *JAMA Psychiatry* studied this self-help intervention in comparison to escitalopram among 208 adults and found that both approaches were similarly effective, with fewer stopping therapy (0% versus 8%) and fewer reporting an adverse event (15.4% versus 78.6%) [24].

Early intervention can prevent the progression to clinical depression in young people. A 2012 Cochrane review analyzed 53 studies representing 14,403 people and determined that a range of early interventions have evidence of success [33]. The authors concluded, “There is some evidence from this review that targeted and universal depression prevention programmes may prevent the onset of depressive disorders compared with no intervention…The persistence of findings suggests that this is real and not a placebo effect [33].”

### Connection to Screening-Informed Higher Levels of Care

Broad screening of a population served by a health plan, community, or employer can rapidly triage between preclinical mental well-being concerns or find those at high-risk of clinical diagnosis for whom further diagnostic intervention and treatment may be appropriate. Linking to resources is then based on one’s screening result and the resources available in the setting of the interaction. This may mean connecting to self-help resources for the 75% of those with a concern who choose to work on it on their own, and referral to resources that a health plan, community, or employer has available for those who reconsider accessing clinical care and for those at higher risk. Digitally enabled screening also facilitates data-informed intake by bringing screening data into the clinical care process.

## Case Example: Monterey County

Monterey County Behavioral Health (MCBH), CA, collaborated with the State of California and a mental health and well-being resource platform (CredibleMind, Inc. Sausalito, CA) to create WellScreen Monterey, an internet-based screening tool covering eight mental health areas—anxiety, depression, postpartum depression, posttraumatic stress disorder, eating disorders, bipolar disease, psychosis, and substance misuse. The tool uses validated scales, and the assessment is available to anyone with internet access. This changed the traditional paradigm of residents needing to visit a county office and deal with paperwork to determine eligibility or to access services. Instead, residents access the platform by computer, tablet, or smartphone, quickly see screening results showing their risk levels, find out if they are eligible for MCBH services, and are offered options for help.

The platform connects users to evidence-based self-help resources, psychoeducational content, and county behavioral health resources, including higher levels of care. Clinical caregivers can access users’ screening results with permission via a provided code.

In the first year of operation, the platform nearly quadrupled the total number of people who accessed county mental health support, from 13,150 clients in 2021‐2022, adding 35,998 WellScreen users. The platform created 8,525 new screenings for MCBH (average 23 screenings/day). Usage data reveal that the vast majority of users are not currently receiving treatment—84% (3781/4502) reported they are not taking medication, 86% (3872/4502) reported that they were not receiving treatment or services for mental health or substance use, and 95% (4277/4502) reported that they have never received services offered via MCBH.

WellScreen Monterey outcome data identified that the majority of people taking assessments have moderate to severe symptoms and would benefit from the provided psychoeducation and access to resources. For anxiety, 57% (2571/4510) scored in the moderate to severe range and 43% (1939/4510) in the mild to minimal range. Similarly, moderate to severe symptoms occurred in 59% (2661/4510) of the participants screened for depression, 49% (2207/4505) for posttraumatic stress disorder, and 47% (2137/4509) for eating disorders. Self-reported satisfaction was high, with 368 of 396 respondents (92.9%) indicating that use of the platform was “helpful.” The digital screening tool effectively identified a significant number of people who have an especially high risk, can benefit, who are not already plugged in to any intervention, and for whom resources could now be offered.

## Conclusion

The alarming rise in the prevalence of mental health challenges is neither inevitable nor irreversible. It is time to meet the growing need with a widely available, proactive population mental health paradigm. By combining high-access digital intervention, proactive community-wide screening, wide access to expert-vetted, evidence-based self-care, and timely low-friction referral to available care resources, we can reduce both suffering and cost, ease pressure on over-taxed systems of care, and create better mental health and well-being at the individual and community-wide levels.

## Abbreviations

MCBH: Monterey County Behavioral Health
